# Do Women Using Long-Acting Reversible Contraception Reduce Condom Use? A Novel Study Design Incorporating Semen Biomarkers

**DOI:** 10.1155/2011/107140

**Published:** 2011-08-07

**Authors:** Maria F. Gallo, Lee Warner, Denise J. Jamieson, Markus J. Steiner

**Affiliations:** ^1^Division of Reproductive Health, National Center for Chronic Disease Prevention and Health Promotion, Centers for Disease Control and Prevention, Atlanta, GA 30341-3724, USA; ^2^Clinical Sciences Division, FHI 360, Durham, NC 27713, USA

## Abstract

Long-acting reversible contraceptive (LARC) methods are highly effective against pregnancy. A barrier to their widespread promotion can include the concern they will lead reduced condom use and, thus, will put couples at higher risk for sexually transmitted infections (STIs). We review evidence from previous studies of condom “migration” associated with the use of LARC and propose a novel study design to address the two main methodological issues that have limited these earlier studies. Namely, we propose to use a randomized controlled trial design and to use a biological marker of semen exposure for measuring changes in condom use.

## 1. Introduction

Because long-acting reversible contraception (LARC) methods have very high efficacy and low burden to users after their initiation, the American College of Obstetricians and Gynecologists recommends that women be offered LARC as first-line contraceptive methods [1]. These methods, which include intrauterine devices (IUD) or systems (IUS) and contraceptive implants, avoid the need for frequent visits for resupply, are highly cost effective, and allow a rapid return to fertility after their removal. However, while LARC methods are extremely effective against pregnancy, they offer no protection against the transmission of HIV and other sexually transmitted infections (STIs). Thus, policy makers, funders, and providers might hesitate to support and promote the use of LARC, in part, because of concerns that women who initiate their use (or their male partners) could be less motivated to use condoms [2, 3]. Because women using LARC essentially are no longer at risk of pregnancy, they may choose not to use condoms or may no longer be able to convince their partner to use condoms solely for disease prevention. Because condoms, used consistently and correctly, remain the most effective method of protection against HIV/STIs for sexually active individuals [4, 5], changes in condom use patterns from the introduction of LARC could put women and their partners at increased risk of infection. Similar concerns about condom “migration” related to the introduction of other interventions, such as microbicides, circumcision, and preexposure prophylaxis for HIV prevention, also have been raised [6, 7].

### 1.1. Literature on Condom “Migration”

The available evidence—albeit limited—supports the possibility that LARC use could lead to reduced condom use. A multivariable analysis of a nationally representative survey of women in the US found that condom use was lower for women using injectables, intrauterine devices or implants compared to women using oral contraception [8], and a survey of reproductive-aged women in Baltimore revealed a significantly lower prevalence of condom use among implant users than nonusers [9]. Similarly, a study among Hispanic and African-American female adolescents in Manhattan found condom use in the prior month was lower among women relying on the injectable or implant compared to those using condoms only [10]. A case-control study of adolescents in Texas found that condom use among implant users was lower than among oral contraceptive users [11].

Three prospective cohort studies also have evaluated the association between LARC and condom use. A study of adolescents in San Francisco found that women who chose to use implants reported less condom use after two years of followup than at baseline [12]. Condom use among implant users was also lower than among those who chose to use oral contraception or condoms alone. In a study conducted in three large urban hospitals of 1,073 women initiating use of implant or injectable contraception, consistent condom use within the past three months declined after one year of followup [13]. Only one study, conducted among 98 postpartum adolescents in Pennsylvania who chose to use either implants or oral contraception, failed to find differences between groups in the levels of condom use reported [14]. 

Prior studies on condom migration suffer from two main methodological weaknesses: reliance on self-reports of condom use and lack of randomization. Participant reports of condom use could be inaccurate for many reasons, including recall bias or—especially in intervention studies in which condoms are heavily promoted or distributed—social desirability bias [15–18]. Studies that have detected biological markers of semen (e.g., prostate-specific antigen [PSA] or y-chromosome DNA) in vaginal fluid specimens collected from women who reported no recent exposure suggest substantial underreporting of unprotected sex [19–24]. Furthermore, misreporting of unprotected sex might not be distributed randomly across a study population, as suggested by a recent study that assessed predictors of discordance between spermatozoa detection and self-reported lack of unprotected sex [25]. For example, if the misreporting of condom use were to differ with respect to the use of other contraceptive methods, the conclusions reached by previous studies on condom migration could be misleading.

The second—and perhaps more serious—limitation of past studies involves the lack of randomization for assigning women to method use. Women who choose different contraceptive methods often vary by their characteristics [26]. Confounding by indication could occur if the women who choose to use LARC methods (or the women whose providers promote LARC methods to) are predominantly those at low risk of HIV/STIs and, thus, infrequent users of condoms. Those who opt to initiate a LARC method may have inherently different patterns of condom use than those who choose not to use the method; thus, earlier studies could be biased as a result of systematic differences between groups. Few studies on the relationship between use of LARC and condoms have attempted to control for these differences and thus are subject to confounding; moreover, controlling for these factors would likely be difficult as not all factors influencing condom use (e.g., perceived HIV/STI risk, ability to negotiate condom use with partner) are known or easily measured.

## 2. Randomized Controlled Trial Using a Semen Biomarker

**Figure 1:**
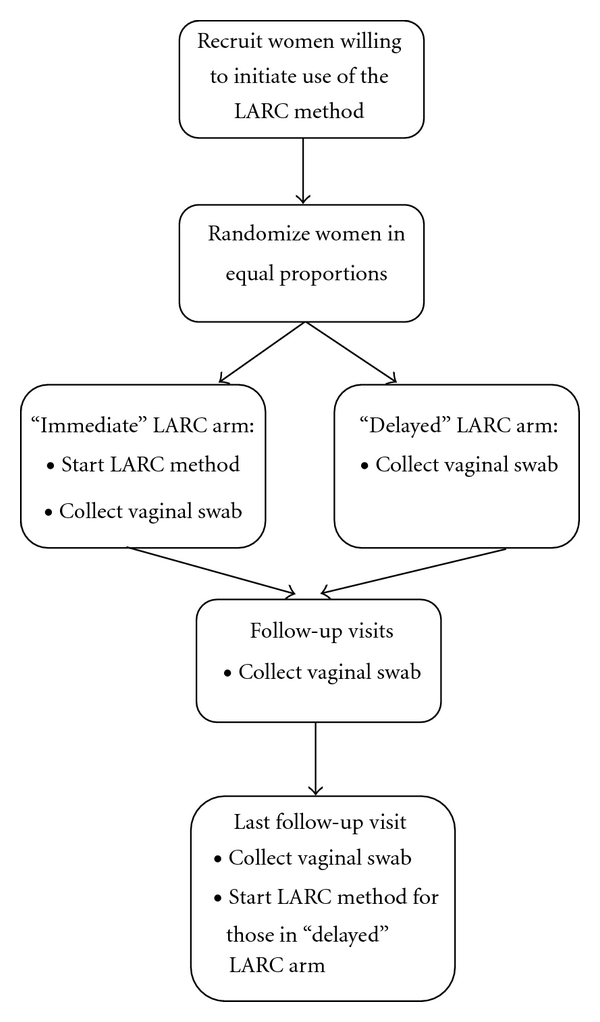
Proposed study schema.

We propose a randomized design for studying the effect of introducing a specific type of LARC method (i.e., IUD, IUS, or contraceptive implant) on condom use that addresses both major methodological weaknesses inherent in the previous studies. Under this design, women at risk of pregnancy without contraindications to the method [27, 28] would be randomized to one of two groups: (1) “immediate” start of the LARC method or (2) “delayed” start of the LARC method (i.e., at completion of their last study follow-up visit) (Figure 1). Participants would be asked to complete an enrollment visit and follow-up visits scheduled at regular intervals during the period (e.g., 6–12 months) following enrollment. Vaginal specimens would be collected—either by swabbing by the participant herself or by a study provider—at all study visits for testing for PSA, and participants would be counseled on correct and consistent use of condoms. The study would assess whether use of the LARC method affects the number of unprotected sex acts (i.e., without a condom) as measured by the detection of an objective biomarker of unprotected intercourse, such as PSA, in the vaginal swab.

PSA, a glycoprotein produced by the prostate gland and secreted into the seminal plasma, was first identified by forensic scientists who were trying to find a substance in seminal fluid to facilitate the investigation of rape cases [29]. More recently, PSA has been used in contraception and HIV/STI research to assess recent (i.e., within the previous 48 hours) exposure to semen [30–33]. While several laboratory assays can be employed to quantify PSA levels or other biomarkers of semen exposure (e.g., Y-chromosome DNA), they require specialized laboratory equipment and training. More recently, a low-cost, rapid test that can be used by laboratory technicians without specialized training has been showed to provide reliable and valid semiquantitative measures of PSA and, thus, makes the use of PSA as a semen biomarker feasible in a wide range of research settings [34].

## 3. Challenges to Study Design

Ideally, such a study would be conducted in a site in which the women normally do not have access or are naïve to use of the specific LARC method. Participants would be randomized to receive the LARC method at enrollment or after the end of followup. Precedent for this type of design in which the introduction of the intervention is delayed in the control arm until the end of study participation can be found, for example, in the recent trials on the effect of circumcision on HIV acquisition in which men were randomized to immediate or delayed circumcision [35]. Ethically, investigators cannot withhold a LARC method (even for a limited interval) from women in settings where the normal standard of care allowed for its provision. Consequently, depending on the availability of the LARC method, this study design might not be feasible in all settings.

Another limitation is the potential for the request for swab collection to influence participant behavior. Women could either be asked for their consent to test their collected vaginal specimens for PSA at time of enrollment or, retroactively, at their last study visit. Although a randomized controlled trial did not find evidence that the advance knowledge of testing for PSA caused women to report more unprotected sex [36], such knowledge still could cause women to modify their behavior (e.g., abstain from unprotected sex before study visits). Asking for consent for PSA testing retroactively circumvents the possibility of behavioral change from swab collection; however this approach requires another reason to justify swab collection during the study (e.g., as an opportunity for STI testing) and would risk losing data from women who do not return for their last visit or who decline to consent to the testing. Regardless of the timing of the consenting process for PSA testing, women simply might tend to change behavior in general before attending study visits; for example, women may abstain from sex before a scheduled pelvic examination due to hygienic concerns.

Any study of risk compensation will have problems with generalizability. Even if the proposed study does not demonstrate evidence of condom migration from the initiation of the contraceptive implant, this would not preclude its occurrence in other settings or with other populations. The effect of adopting a LARC method on participant levels of condom use could vary by numerous factors, including participant characteristics, participant perceived risk of STIs, quality of provider counseling and patient-provider rapport, and social norms surrounding condom use. Study findings from one setting cannot be assumed to be generalizable to other populations.

## 4. Conclusions

The proposed study design addresses two methodical weaknesses (i.e., reliance on self-reported data and lack of randomized design) that have limited the interpretation of findings from previous studies on this topic. More broadly, the detection of semen biomarkers could be applied to research on condom migration associated with other contraceptive methods and with HIV-prevention interventions, including male circumcision and oral or topical microbicides. Given that the occurrence of condom migration from the introduction of LARC methods or HIV-prevention interventions could vary by study populations, the utility of testing for PSA might be even more useful for on-going, population-level surveillance of changes in condom use patterns. This surveillance could provide accurate information with which to identify subgroups in a setting that might benefit from more intensive condom counseling. Also, by monitoring the frequency of PSA detection in a population, providers could evaluate and compare the effectiveness of different methods of counseling on condom use. Researchers should consider incorporating testing for semen biomarkers routinely into studies as well as surveillance requiring measures of unprotected sex.
